# Effects of Freeze–Thaw Cycles on the Structures and Functional Properties of *Clitocybe squamulosa* Protein Isolates

**DOI:** 10.3390/foods12152948

**Published:** 2023-08-04

**Authors:** Lijing Xu, Xin Wang, Yaping Xu, Junlong Meng, Cuiping Feng, Xueran Geng, Yanfen Cheng, Mingchang Chang

**Affiliations:** 1 College of Food Science and Engineering, Shanxi Agricultural University, Taigu 030801, China; 2 Shanxi Key Laboratory of Edible Fungi for Loess Plateau, Taigu 030801, China; 3 Shanxi Engineering Research Center of Edible Fungi, Taigu 030801, China

**Keywords:** *Clitocybe squamulosa* protein isolate, freeze–thaw cycle, physicochemical and functional properties

## Abstract

Changes in the functional properties and structures of *Clitocybe squamulosa* protein isolate (CSPI) in the process of freeze–thaw (F–T) cycles were explored. Remarkable alterations and the reduced content of protein ordered structure were revealed through structural analysis of CSPI after F–T treatments. The surface hydrophobicity and free sulfhydryl content of CSPI first increased and then decreased. However, after the F–T treatments, the carbonyl content of CSPI continued to increase. Similarly, the water holding capacity (WHC), oil holding capacity (OHC), and solubility of CSPI all declined as the number of F–T cycles increased. The foaming properties and emulsifying properties of CSPI were significantly improved and reached maximum values after three F–T cycles. CSPI undergoing two F–T cycles showed the highest digestibility, maximum polypeptide content, and highest DPPH and ·OH-radical-scavenging activities. The ·OH-radical-scavenging activities and reducing power of the gastrointestinally digested CSPI had the highest value after one F–T cycle. Therefore, it has been demonstrated that F–T treatments could be a residue-free and cost-effective tool for improving mushroom protein functional properties.

## 1. Introduction

Proteins are one of the vital components of the human diet and contribute to the physiological functions of the human body. Food proteins have abundant and diverse sources, including animals, plants, algae, and microorganisms. The current protein market is dominated by milk, egg, and legume proteins. With the increasing demand and adverse environmental impacts of proteins from conventional sources, more attention has been given to various nonconventional sources [1].

Edible mushrooms are healthy alternatives to high-quality proteins in food products, as they are rich in proteins (from 18.87% to 36.96% of dry weight) containing all essential amino acids [2]. In addition, edible mushrooms have high nutritional value and healthy functions in the human body. Hence, exploring novel protein sources from edible mushrooms is important for product formulation and protein fortification.

*Clitocybe squamulosa*, also called “Taimo” by local people, is a wild edible mushroom pervasive in Wutai Mountain, Shanxi Province, China. *C. squamulose* contains ample nutrients, such as polysaccharides, proteins, and crude fibers [3]. Due to its attractive flavor, distinctively rich taste, and a variety of bioactive compounds, *C. squamulose* is popular with local people and tourists. As a local proverb goes, “A family has its soup, ten neighbors smell its tantalizing aroma”. To date, most of the publications on *C. squamulose* have focused on its polysaccharides instead of other nutrients, such as proteins. As a new source of high nutrient proteins, *C. squamulose* has potential applications in the food production industry. Therefore, developing *C. squamulose* protein isolate (CSPI) and expanding its utilization in the food production industry are necessary.

At present, researchers have proposed various modification approaches (such as physical, chemical, and enzymatic treatments) to improve the functional and physicochemical properties of proteins [4]. Compared with chemical and enzymatic treatments, the major advantage of physical treatment is that it is residue-free, which ensures the purity of the final product. Freezing treatment is a moderate physical method that can lead to possible structural modifications and affect functionality properties with minimum impact. This is because protein structures change when freezing and protein molecules are rearranged [5]. It was reported that cross-linking and oxidation of proteins were caused by multiple F–T cycles, leading to changes in protein structures and internal water mobility [6]. Moreover, repeated F–T cycles can also effectively promote the foaming properties of egg white proteins and albumen proteins, except for ovalbumin [7]. In addition, the emulsification of egg yolk and soy protein isolates can be greatly improved by carrying out F–T treatments [8,9]. The amount of improvement in emulsification may be connected with the F–T cycle number. There is evidence to show that when prepared through PPI via 3 F–T cycles, the emulsion shows optimal performance in emulsification [10]. Furthermore, physical modification was also associated with an increase in in vitro digestibility [11]. Therefore, F–T treatments can probably provide a novel opportunity to improve the quality of mushroom proteins.

However, to the best of our knowledge, there is no published research on CSPI, especially its structural and functional properties. Therefore, this research aimed to explore how repeated F–T treatments affect the structures and functional characteristics of CSPI. Free sulfhydryl content, carbonyl content, surface hydrophobicity, scanning electron microscopy (SEM), and Fourier transform infrared spectroscopy (FTIR) were conducted to characterize structural changes. The solubility, OHC, WHC, emulsifying, and foaming properties of the proteins as well as average particle size and zeta potential of emulsions were measured to reveal the effects of multiple F–T treatments on the emulsification characteristics of CSPI. Next, in vitro gastrointestinal digestion (IVGD) and antioxidant capacities were investigated. This research will lay a theoretical foundation for CSPI, and enable evaluation of its potential applications in food emulsions.

## 2. Materials and Methods

### 2.1. Materials and Reagents

Fruiting bodies of *C. squamulosa* were obtained from the Shanxi Engineering Research Center for Edible Fungi (Jinzhong, Shanxi, China).

Pepsin from the porcine stomach and 1,1-diphenyl-2-picrylhydrazyl (DPPH) were purchased from Sigma Chemical Co. (St. Louis, MO, USA). Other chemical reagents were all analytically pure.

### 2.2. CSPI Preparation

Preparation of CSPI followed Feng’s approach with some modifications [10]. After crushing and sifting dry samples via a 100-mesh screen, the resulting powder was blended with n-hexane (1:5, *w*/*v*), followed by 40 min of extraction at room temperature to remove lipids. The supernatant was dried in a fume hood. Deionized water was then added to defatted samples at a ratio of 1:30 (*w*/*v*). After using 1 M NaOH to adjust the pH to 11, the mixture was treated via ultrasonication for 60 min. After 10 min of centrifugation of the suspension liquid at 4500× *g*, 1 M HCl was added to the supernatant to adjust the pH to 3.6, followed by centrifugation at 6500× *g* for 15 min. After dispersing the precipitate in deionized water, 1 M NaOH was added to adjust the suspension liquid to neutral. After lyophilizing the finally obtained CSPI solution, the resulting powder was preserved at −20 °C for later use.

### 2.3. F–T Treatment

Deionized water was used to redissolve the lyophilized proteins, thus obtaining a final concentration of 20 mg/mL. Subsequently, 2L of CSPI suspension liquid were preserved in 25 polyethylene vials at −20 °C for 24 h and thawed at room temperature for 5 h. Five polyethylene vials were selected and named as one F–T cycle (F–T1) CSPI. The other 20 vials were frozen again for the next F–T treatment. There was no time interval for each F–T treatment repetition. This F–T treatment were carried out 1–5 times, which were named F–T1, F–T2, F–T3, F–T4, and F–T5, respectively. Samples without F–T treatment were labeled as F–T0 CSPI.

### 2.4. Determination of Free Sulfhydryl Content

Free sulfhydryl contents of the CSPI were evaluated by the means of Xiong with slight modifications [12]. The 0.1 M Tris-Gly buffer (pH 8.0, comprising 4 mM EDTA, 0.086 M Tris and 0.09 M glycine) was adopted to prepare dispersed CSPI samples (3 mg/mL) experiencing various F–T cycles. Then, Ellman’s reagent (30 μL, 4 mg/mL 5,5′-dithiobis 2-nitrobenzoic acid (DTNB) in Tris-Gly buffer) was added. After 1 h of incubation of this mixture in the dark at room temperature and 15 min of centrifugation at 10,000× *g*, the supernatant absorbance was measured against the reagent blank at 412 nm.

### 2.5. Determination of Carbonyl Content

Determination of carbonyl contents was performed according to Liu’s approach with slight modifications [13]. CSPI sample dispersions (20 mg/mL) undergoing various F–T cycles and consisting of 2 M HCl were blended with 10 mM DNPH (consisting of 2 M HCl) at a ratio of 1:3. After standing at room temperature for 1 h, trichloroacetic acid (TCA, 1 mL, 20%, *w*/*v*) was adopted to precipitate a 4 mL mixture. After centrifuging the mixtures at 12,000× *g* for 10 min, washing precipitates 3 times using a mixed solution consisting of anhydrous ethanol and ethyl acetate (1:1, *v*/*v*) and 3 mL using guanidine hydrochloride (6 M) to dissolve precipitates, researchers measured the absorbance at 370 nm.

### 2.6. Determination of Surface Hydrophobicity

Chen’s method (with some modifications) was used to evaluate the surface hydrophobicity of CSPI samples [14]. CSPI sample dispersions (5 mg/mL) experiencing various F–T cycles were prepared using phosphate buffer (20 mM, pH 7.0). Then, 200 μL of 1 mg/mL BPB (bromophenol blue) was added to 1 mL of CSPI sample solution. After 2 h of room-temperature reaction, 15 min of centrifugation of mixtures was performed at 6000× *g*. This was followed by absorbance measurement of supernatant against phosphate buffer blank at 595 nm.

The amount of BPB bound below was used to represent the surface hydrophobicity index:

$$\mathrm{BPB}\;\mathrm{bound}\;(µg)\;=\frac{200\times (A_{0}-A_{1})}{A_{1}}$$

where A_0_ and A_1_ separately represent absorbances at 595 nm of CSPI samples not mixed and mixed with BPB.

### 2.7. FTIR

An FTIR spectrometer (Bruker Tensor 27, Bruker Optics, Ettlingen, Germany) was employed to determine the spectra of CSPI undergoing various F–T cycles. After mixing with KBr (1:100), CSPI was compressed as a thin tablet to scan at wavelengths of 4000–400 cm^−1^. Peakfit Version 4.12 (SPSS Inc., Chicago, IL, USA) software was adopted to analyze the FTIR spectrum data and the protein secondary structure.

### 2.8. SEM

SEM was used to scan the morphologies of gold-sputtered CSPI under a JEM-6490LV scanning electron microscope (JEOL, Tokyo, Japan).

### 2.9. Functional Properties

#### 2.9.1. Solubility

Zhao’s procedure (with some modifications) was referred to when determining the solubility of CSPI [15]. Deionized water was adopted to prepare CSPI sample dispersions (2 mg/mL) undergoing various F–T cycles and to adjust the mixture to neutral. CSPI sample dispersions were oscillated for 5 min, followed by 15 min of centrifugation of mixtures at 6000× *g* and separation of supernatant. The protein contents of the supernatant and suspensions were detected following the Coomassie brilliant blue method and the Kjeldahl method for nitrogen determination, respectively. The equation below was used to calculate protein solubility:

$$\mathrm{Solubility}\;(\%)=\frac{m_{1}}{m}\times 100$$

where m and m_1_ separately denote the protein contents in suspensions and supernatants.

#### 2.9.2. WHC and OHC

Determination of the OHC and WHC of CSPI was performed according to previous research with slight modifications [15]. CSPI samples (0.2 g) treated by different F–T cycles were placed into preweighed centrifuge tubes with deionized water (5 mL) or soybean oil (10 mL) and mixed by vortexing. After being centrifuged at 6500× *g* for 15 min, excess water or oil was removed with caution using absorbent paper.

Calculation formulas for WHC and OHC were expressed below:

$$\mathrm{WHC}(\%)\;=\frac{{m_{2}-m}_{1}}{m}$$

$$\mathrm{OHC}(\%)=\frac{{m_{2}-m}_{1}}{m}$$

where *m*, *m*_1_, and *m*_2_ denote the masses of the samples, samples and centrifuge tubes, and precipitates and centrifuge tubes, respectively.

#### 2.9.3. Foaming Capacity and Foaming Stability

Analysis of foaming capacity (FC) and foaming stability (FS) followed procedures proposed by Kim et al. [16]. CSPI dispersion (25 mL of 5 mg/mL) experiencing various F–T cycles was placed in a centrifuge tube for 2 min of homogenization at 10,000 rpm via a high shear dispersing emulsifier (FA25, FLUKO, Shanghai, China), during which, volumes of dispersions were recorded. After preservation at room temperature for 10 min, volumes were measured again. Calculation formulas for FC (%) and FS (%) are:

$$\mathrm{FC}(\%)\;=\frac{V_{0}}{V}\times 100$$

$$\mathrm{FS}(\%)\;=\frac{V_{10}}{V}\times 100$$

where *V* and *V*_0_ separately denote the volumes of unhomogenized and homogenized dispersions and *V*_10_ is the foam volume after being stored for 10 min.

#### 2.9.4. Emulsifying Activity Index and Emulsifying Stability Index

Measurement of the emulsifying activity index (EAI) and emulsifying stability index (ESI) followed the approach reported by Zhao et al. with some amendments [15]. Homogenization of CSPI dispersions (25 mL, 20 mg/mL) undergoing various F–T cycles and soybean oil (10 mL) was performed at 10,000 rpm for 2 min by a high shear dispersing emulsifier (FA25, FLUKO, China). Then, the emulsion (50 µL) was removed at the bottom of the beaker, and SDS solution (10 mL, 0.1%, *m*/*v*) was aspirated to mix them adequately. This was followed by absorbance measurement at 500 nm before and after 10 min of preservation with SDS solution as a blank. Calculation formulas for EAI and ESI were:

$$\mathrm{EAI}(m^{2}/g)=\frac{2\times 2.303\times N\times A_{0}\;}{C\times \varphi \times L\times {\;10}^{4}}$$

$$\mathrm{ESI}(\min)=\frac{A_{0}\times 10}{A_{0}-A_{10}}$$

where *N* represents the dilution factor; *A*_0_ and *A*_10_ denote absorbances before and after storage for 10 min; and *C*, *φ*, and *L* represent the protein concentration, volume fraction of oil, and optical path, respectively.

#### 2.9.5. Average Particle Size and Zeta Potential of Emulsions

After various F–T cycles, 5 mL CSPI dispersions (1 mg/mL) underwent 15 min of centrifugation at 6500 rpm, followed by 2 min of homogenization of 20 µL supernatant and 5 mL soybean oil at 10,000 rpm with a high shear dispersing emulsifier (FA25, FLUKO, Shanghai, China). A membrane filter with a pore size of 0.45 μm and an S90 Zetasizer Nano instrument (Malvern Instruments Ltd., Worcestershire, UK) were separately used to filter and detect the emulsion.

### 2.10. IVGD

IVGD was performed using a method proposed by Cho with a modification [17]. To investigate this further, acquired ME was subjected to air-drying and milling via a 40-mesh screen. IVGD of CSPI powder (0.5 g) treated in different F–T cycles was simulated by dissolving them in artificial simulated gastric fluid. After using 1 M HCl to adjust the pH of CSPI dispersions (10.0 mg/mL) to 3.0 and incubating them at 37 °C for 2 h, the resulting digests were terminated by using 1 M NaOH to adjust the pH to 7.0 and boiling for 10 min. The supernatant of gastric digested samples was collected for analysis after being centrifuged for 15 min at 6500 rpm. The specimens were referred to as CSPI-S. After using artificial intestinal juice to mix with gastric digested samples at 1:1 (*v*/*v*) and adjusting the pH of the mixture to 7.0, the mixture was incubated at 37 °C for 2 h. Digestion was terminated by inactivating enzymes through 10 min of boiling of the mixture. Afterwards, the supernatants of intestinally digested samples centrifuged for 15 min at 6500 rpm were collected for analysis. The samples were labeled CSPI-I. To determine the in vitro digestibility of CSPI, 10% (*w*/*v*) trichloroacetic acid solution was added to specimens (1:1, *v*/*v*) and incubated for 1 h. After 20 min of centrifugation at 8000 rpm, the researchers collected the precipitates. The Kjeldahl method of nitrogen determination was adopted to estimate the protein content.

The calculation formula for the in vitro digestibility of CSPI was:

$$\mathrm{Digestibility}(\%)\;=\frac{\mathrm{Total}\;\mathrm{protein}\;\mathrm{content}-\mathrm{Protein}\mathrm{in}\mathrm{precipitates}}{\mathrm{Total}\;\mathrm{protein}\;\mathrm{content}}\times 100$$

### 2.11. The Polypeptide Content

After mixing digestive products with 10% TCA (1:1, *v*/*v*), the mixture was allowed to stand for 15 min. Then, centrifuged supernatant (10,000× *g*, 5 min) was mixed with 5% TCA to 10 mL, followed by addition of biuret reagent to mixtures (4:1, *v*/*v*), 15 min of storage, and measurement of supernatant absorbance centrifuged at 10,000× *g* for 15 min at 540 nm. The polypeptide content was detected in the samples by comparing standard curves for bovine serum protein.

### 2.12. In Vitro Antioxidant Activities

For digestive products, previous approaches were employed to determine the DPPH scavenging activity, OH-radical-scavenging activity, and reducing power [17]. An ABTS test kit was utilizedc to estimate the ability to scavenge ABTS free radicals.

### 2.13. Statistical Analysis

All experiments were replicated three times. The results were expressed as means and standard deviations (SD) and were analyzed using SPSS software (version 16.0, SPSS, Inc., Chicago, IL, USA). Analysis of variance (ANOVA) was performed (*p* < 0.05) between means using the Duncan’s multi-range test.

## 3. Results and Discussion

### 3.1. Free Sulfhydryl Content

**Figure 1:**
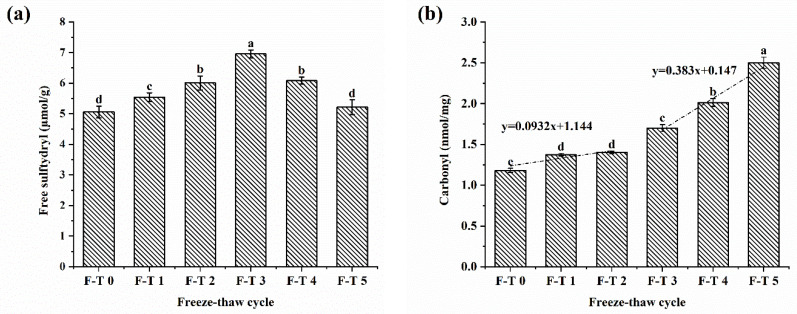

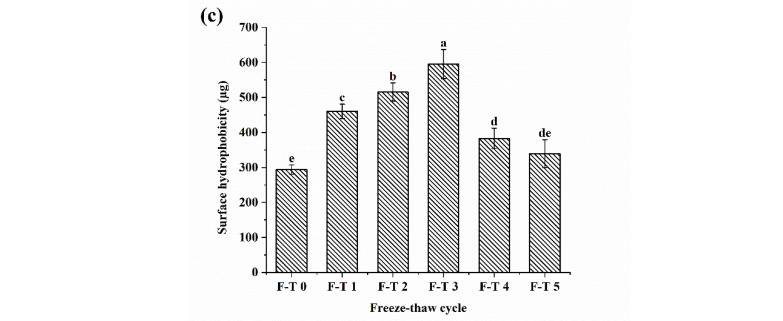
Influence of different F–T cycles on the free sulfhydryl (**a**), the carbonyl (**b**), and surface hydrophobicity (**c**) of CSPI. The different letters indicate statistically significantly in the CSPI with different F-T cycles (*p* < 0.05).

The disulfide bond plays an important role in maintaining the protein structure. Hence, variation in tertiary structures and oxidation of proteins could be reflected by the contents of free sulfhydryl groups [10]. As shown in Figure 1a, when the number of FT cycles increased, the free sulfhydryl content rose at first, followed by a decline (*p* < 0.05), and it reached a maximum of 6.95 ± 0.13 μmol/g (FT = 3). The results indicated that the F–T cycles might break the disulfide bonds of proteins. Due to the weakness of covalent disulfide bonds, ice recrystallization and water redistribution during F–T cycles might break the cause of the disulfide bonds, which increased the free sulfhydryl group content. Following too many F–T cycles (F–T = 3–5), the free sulfhydryl groups were oxidized into novel disulfide bonds, resulting in decreased contents of free sulfhydryl. Similarly, the contents of free sulfhydryl groups of peanut protein isolates subjected to F–T cycles also first increased and then decreased [10].

### 3.2. Carbonyl Content

Studies have found that ROS-induced damage can cause the production of protein carbonyl groups. Therefore, the protein carbonyl content is among the critical parameters for the oxidation of proteins [10]. The protein carbonyl content of CSPI samples changed with increasing F–T cycles (Figure 1b). During the F–T treatments, the carbonyl content in CSPI increased significantly (*p* < 0.05). It first increased slowly and then increased rapidly. The results indicated oxidization of CSPI via F–T cycles, which conformed to previous results of a continuous increase in the carbonyl content of peanut protein isolate after F–T treatments [10]. The freezing and thawing cycles led to ice recrystallization and ice crystal growth. An increased volume of ice crystals damaged protein structures, causing the formation of reactive oxygen for pro-oxidative components and resulting in increased carbonyl content in proteins [18]. However, the decrease in the rate of carbonyl formation following repeated F–T cycles (3–5) was probably because of the more compact overall structure of CSPI, which was not easy to oxidize.

### 3.3. Surface Hydrophobicity

Surface hydrophobicity directly reflects how much hydrophobic groups of proteins are exposed, indicating changes in the functional properties and conformational structures of proteins [19]. It is an important characteristic of denaturation degree and gelatinization of protein. Figure 1c showed remarkable growth of hydrophobicity to a maximum of 595.35 μg after three F–T cycles, indicating that F–T cycles cause molecular rearrangement and surface exposure of the hydrophobic side-chain group. Meanwhile, the hydrophobicity declined sharply in the late stage of the F–T cycles, probably because of the aggregation of unfolded proteins by oxidation during multiple freezing and thawing cycles. Similar results had also been observed in F–T-treated ovomucoids and peanut protein isolates [7,10]. Moreover, hydrophobicity variation was closely correlated with emulsifying properties, which could explain the trend of emulsion stability and emulsifying activity.

### 3.4. Variation of Secondary Structures

**Figure 2:**
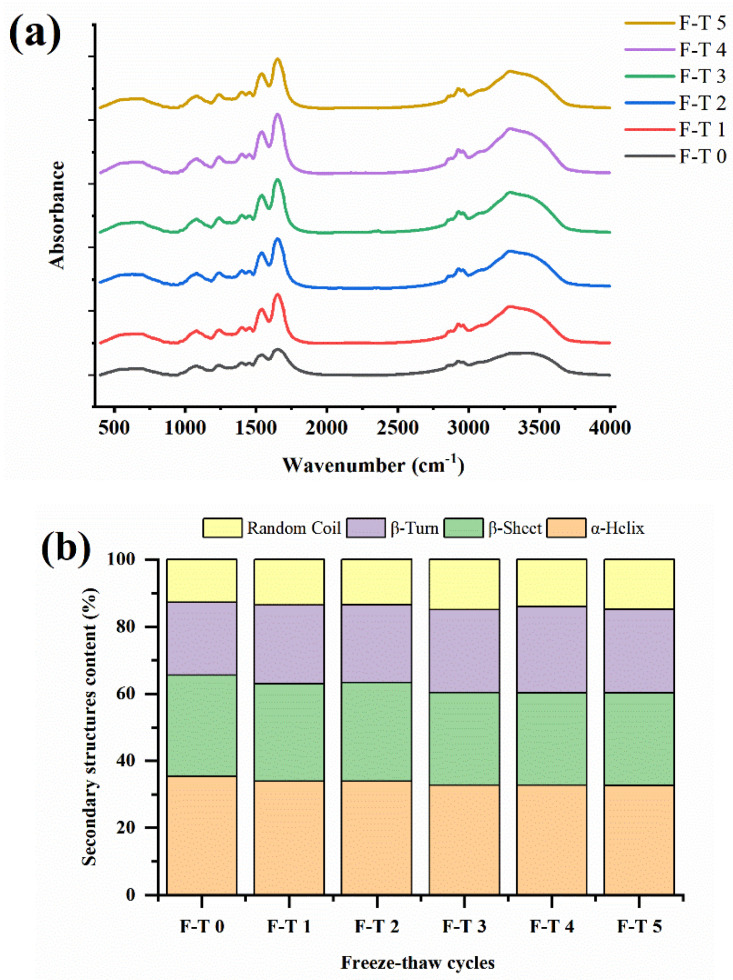
Influence of different F–T cycles on the structure of CSPI. (**a**) FTIR; (**b**) secondary structures content.

Infrared spectra of natural and F–T-treated CSPI samples are shown in Figure 2a. The peaks at 1700–1600 cm^−1^ resulted from amide I bands, which are mainly caused by C=O stretching in proteins. The peaks at 3600–3100 cm^−1^ were probably attributed to N−H stretching. Stronger absorption peaks were found for CSPI samples treated with F–T cycles at 1700–1600 and 3600–3100 cm^−1^.

Figure 2b displayed the contents of secondary structures of CSPI. The α-helix and β-sheet contents accounted for larger proportions of total structures in folded proteins. Both of them were formed and stabilized by noncovalent interactions, mainly hydrogen bonds of N–H and C=O groups [13]. Compared to hydrogen bonds in α-helices, the bonds in β-sheets were between adjacent strands. The relative contents of β-sheets and α-helices gradually decreased when the number of F–T cycles increased. The decrease in the ordered structure resulted in looser and more flexible protein molecules, which showed that the F–T cycles destroyed the intermolecular interactions.

Meanwhile, the content of disordered structures included random coils and β-turns, indicating the probability of proteins undergoing unfolding, dissociation, and rearrangement. The results showed influences of F–T treatment cycles on secondary structures in CSPI. More disordered protein molecules had the expectation to exhibit preferable functional properties.

### 3.5. SEM

**Figure 3:**
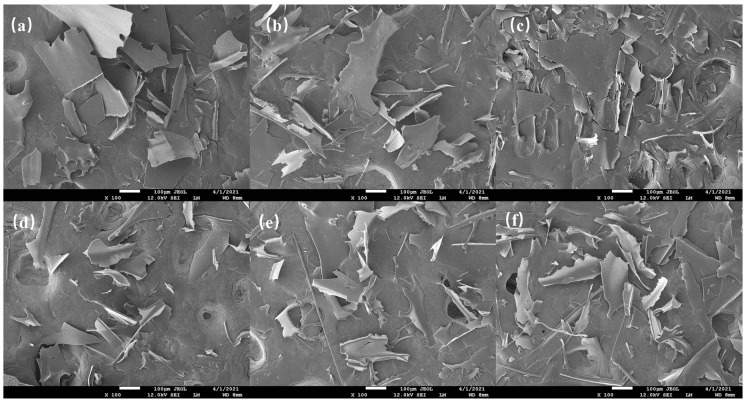
SEM images of CSPI with different F–T cycles. (**a**) F–T 0; (**b**) F–T 1; (**c**) F–T 2; (**d**) F–T 3; (**e**) F–T 4; (**f**) F–T 5.

SEM was adopted to visualize the effects of F–T cycles on CSPI microstructures (Figure 3). The microstructure of CSPI without F–T treatment was characterized by larger flakes and slightly curved. The surface was smooth and compact, with a small notch at the edge, but most of them were complete, and no obvious fine fragments were seen. After the F–T treatments, the size of the flakes decreased, and the irregular gaps at the edge of the flakes increased. The results implied damage of F–T treatments to the structural integrity of CSPI. Three F–T treatments led to the smallest and uniformly distributed flake structures, which was consistent with that of peanut protein isolates [10].

### 3.6. Functional Properties

Functional properties of proteins are significant for the food manufacturing process, imparting sensory and texture properties to food products. In this study, the solubility, WHC, OHC, FC, FS, emulsifying capacity, emulsifying stability of the proteins as well as average particle size and zeta potential of emulsions before and after F–T treatments were investigated.

**Figure 4:**
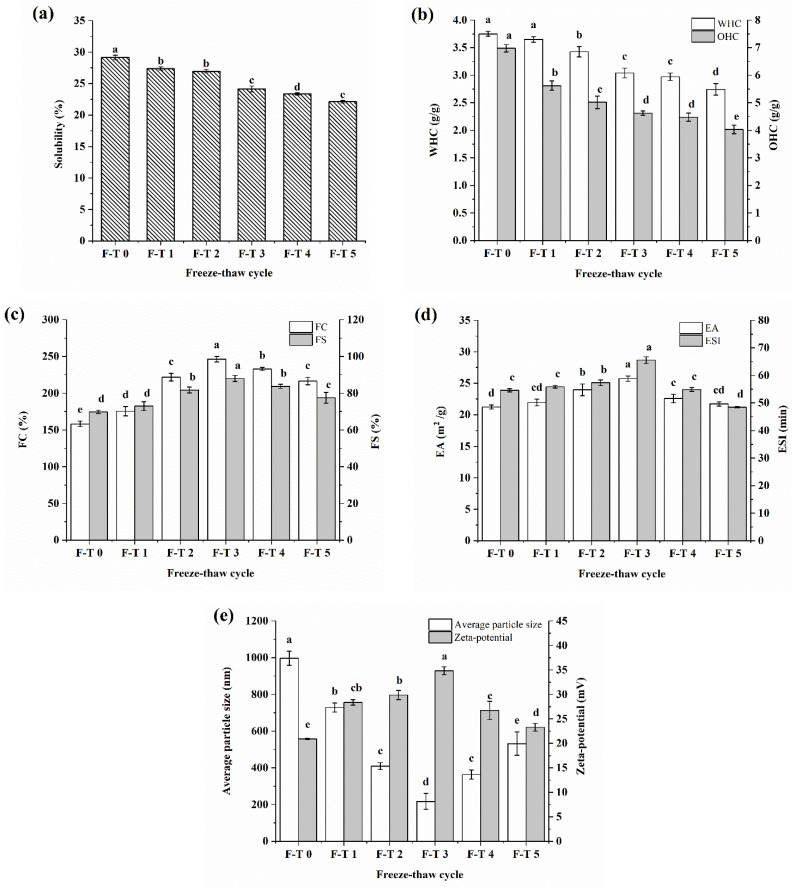
Influence of different F–T cycles on the functional properties of CSPI. (**a**) solubility; (**b**) water holding capacity and oil holding capacity; (**c**) foaming capacity and foaming stability; (**d**) emulsifying activity index and emulsifying stability index; (**e**) average particle size and zeta potential of emulsions. The different letters indicate statistically significantly in the CSPI with different F-T cycles (*p* < 0.05).

Solubility, as a critical protein functional property, influences other functional properties of proteins. It can reflect the aggregate states and denaturation degrees of proteins and affect their utilization during food production [20]. Protein solubility is determined by the sensitive balance of attractive and repulsive intermolecular interactions controlled by variations in conformations. The solubility variation of CSPI before and after F–T treatment was given in Figure 4a. With the growing number of F–T cycles, CSPI solubility gradually decreased, which was caused by denaturation of proteins resulting from ice recrystallization during the F–T cycles. Moreover, constant exposure of hydrophobic groups weakens the binding effect with water molecules and results in reduced solubility. Similarly, the solubility of pea proteins also showed a significant reduction after F–T treatment [5].

WHC is the ability of proteins to retain water throughout processing and storage and reflects the interaction between water and proteins [21]. Figure 4b shows how F–T treatments affect the OHC and WHC of CSPI. Native CSPI showed a WHC of 3.75 g/g, similar to quince seed proteins (4.01 g/g) and soybean protein isolates (4.36 g/g) in previous research [15,21]. For F–T treated CSPI, WHC exhibited a gradual decreasing trend to a minimum of 2.743 g/g. According to the results of surface hydrophobicity, the WHC declined, possibly because hydrophobic side-chain groups were exposed in multiple F–T cycles.

OHC, the amount of oil with direct binding to proteins, affects flavor retention and mouthfeel. The OHC of CSPI decreased from 6.98 to 4.03 g/g as the number of F–T cycles increased. The decrease in OHC was not completely in line with the trend in surface hydrophobicity, since the effect of F–T treatment on the OHC of CSPI was determined by many factors. Generally, proteins with lower surface hydrophobicity tend to have lower lipid absorption rates [15]. Therefore, the decrease in OHC after F–T treatment may be attributed to the aggregation of protein and buried hydrophobic groups of CSPI. Additionally, enhanced disulfide group formation also could decrease the OHC of protein. Although the F–T treatment decreased the OHC, it still showed superior values compared to commercial proteins such as soy protein (1.2 g/g) or milk protein (2.8 g/g) [22]. This indicated that CSPI has potential as a commercial oil binder in food formulations.

Figure 4c illustrates the influences of different F–T cycles on foaming properties. CSPI was found to increase and then declined in FC and FS as the number of F–T cycles increased. At three F–T cycles, maximum FA and FS values of 264.2% and 88%, respectively, were obtained. Such a change trend of foaming properties conforms well to the -SH results, indicating conformation unfolding and rearrangement of CSPI under F–T treatment and enhanced absorbing ability of the air–water interface. In addition, foaming properties were related to surface hydrophobicity because interactions of the air–water interface were enhanced by exposed hydrophobic groups. All of the above findings indicated the function of fewer F–T treatments in enhancing foaming capacities and expanding the usage of CSPI in food production systems.

The emulsifying properties of proteins are the capacity to promote emulsion formation and maintain emulsions to be stable within some time [23]. The emulsifying properties of proteins can be reflected by EAI and ESI. Figure 4d illustrated the effects of various F–T cycles on the ESI and EAI of CSPI. Untreated CSPI exhibited the lowest EAI and ESI. After F–T treatment, both EAI and ESI increased at first and then decreased, reaching maximums of 25.75 m^2^/g and 66.57 min at three F–T cycles, respectively. The results indicate that fewer F–T cycles enable CSPI to greatly improve its emulsifying properties, which were positively associated with surface hydrophobicity. This was because hydrophobic groups are exposed to the protein surface, promoting quick adsorption of proteins to the oil side of interfaces and consequently decreasing the interfacial tension [23]. Feng reported similar observations on the positive correlation between the emulsibility of peanut protein isolates and their surface hydrophobicity [10]. Meanwhile, too many F–T cycles caused proteins to aggregate, and the emulsifying properties of CSPI to decline. Furthermore, it was found that the improvement of emulsifying properties is a result of disordered structures of proteins after F–T treatments.

Figure 4e illustrate the effects of F–T treatments on the zeta potential and average particle size of emulsions. In general, particle size has a direct impact on emulsifying stability. The smaller the dispersed particle sizes are, the more stable the emulsions are. After F–T treatment, the average particle size first decreased, followed by enlargement, reaching a maximum of 216.8 nm at three F–T cycles. The results conformed to the emulsifying properties mentioned above. This phenomenon might be due to the larger adsorption capacity on the oil–water interface because hydrophobic groups are exposed during the F–T treatment. Based on the results regarding the contents of free sulfhydryl and carbonyl groups, CSPI was found to have gradually increased particle size due to oxidation by 3–5 F–T cycles. Therefore, fewer F–T cycles were able to further enhance the emulsification performance of CSPI.

Zeta potential is important for stabilization of particle suspensions because of electrostatic repulsion of particles showing identical electric charges. The zeta potential in CSPI emulsions increased rapidly to 34.8 mV and then decreased to 23.3 mV after multiple F–T treatments. The zeta potential reached a maximum after the third F–T treatment, which was greatly improved compared with untreated CSPI (*p* < 0.05). This indicated that the electrostatic interaction force in the emulsion was enhanced after repeated F–T cycles. Basically, a higher zeta potential (absolute value) of an emulsion contributes to a relatively stable system. This result was in line with the emulsion average particle size data.

### 3.7. Effect of F–T Treatment on IVGD of CSPI

**Figure 5:**
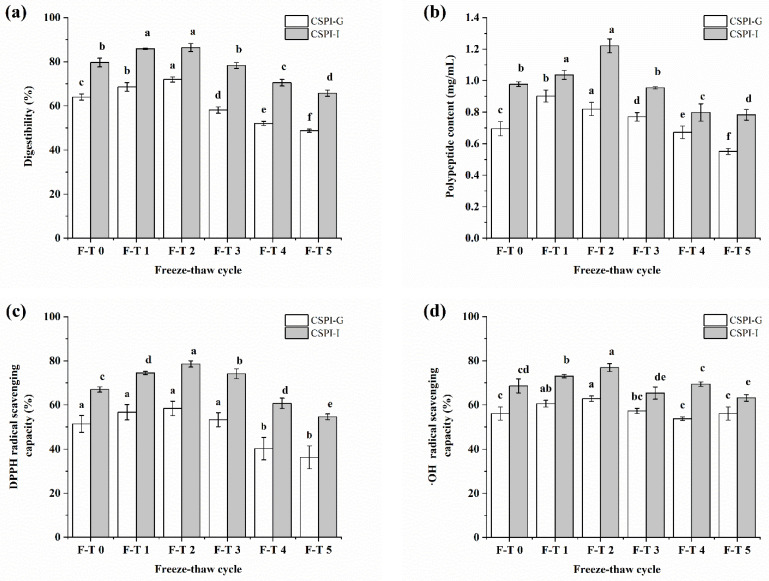

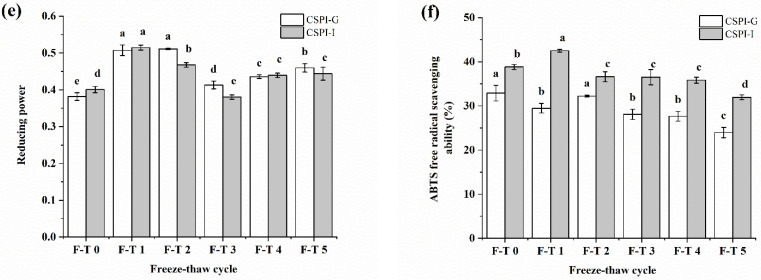
Influence of different F–T cycles on in vitro digestibility (**a**), polypeptide content (**b**), and in vitro antioxidant activities of CSPI during digestion. (**c**) DPPH-radical-scavenging capacity; (**d**) OH-radical-scavenging capacity; (**e**) reducing power; and (**f**) ABTS-radical-scavenging capacity. The different letters indicate statistically significantly in the CSPI with different F-T cycles (*p* < 0.05).

Protein digestibility refers to the degree to which proteins are digested in the body. It is among the important indexes for evaluating the nutritive value of proteins in food. Proteins with higher digestibility values are normally desirable and are more easily absorbed. Figure 5a shows that digestibility grew slowly initially, reached a maximum at the second F–T cycle and then declined quickly in all groups. The protein digestibility was improved after the proper F–T cycles (1–3). This may be explained by molecular unfolding and dissociation as well as rearrangement of proteins because of ice crystal growth during the early stage of F–T cycles. The unfolding structure promoted the binding of digestive enzymes and proteins, resulting in a small increase in the in vitro digestibility of CSPI. The decrease in the digestibility of CSPI may result from the degeneration and reaggregation of proteins due to excessive F–T treatments (3–5 F–T cycles). This result indicated more compact structures of CSPI prepared by multiple F–T cycles than CSPI undergoing fewer F–T cycles. Moreover, the CSPI in the gastrointestinal digestion group was always more digestible than that in the gastric digestion groups.

### 3.8. Polypeptide Content

The effects of F–T treatments on polypeptide content after simulated IVGD are shown in Figure 5b. The trend of polypeptide content was roughly the same as the results of digestibility, which first increased and then decreased during the F–T process. Moreover, the gastrointestinal digestion groups contained a higher peptide content than the gastric digestion groups. This was probably a result of further digestion in the intestinal system. Without F–T treatment, the polypeptide content after gastric digestion and gastrointestinal digestion was 0.69 and 0.98 mg/mL, respectively. The gastric digestion groups had the highest polypeptide content (0.9 mg/mL) after performing one F–T treatment (*p* < 0.05), which then slowly decreased to 0.55 mg/mL. The maximum peptide content of the gastrointestinal digestion group was 1.22 mg/mL after the second F–T cycle. These results indicated the improvement effect of proper F–T cycles (1–3) on the polypeptide content.

### 3.9. Antioxidant Activity

The effects of F–T treatments on the antioxidant activity of CSPI after simulated IVGD were explored. Variations in the radical-scavenging activities for DPPH, OH, and ABTS as well as the reducing power of CSPI in the IVGD process were evaluated (Figure 5). The results suggest the same change trend of antioxidant activity as that of the polypeptide content, which increased first and then decreased during the F–T process. CSPI after two F–T cycles had the highest digestibility, the maximum polypeptide content, and the highest radical-scavenging activities for DPPH and OH. The maximum OH-radical-scavenging activity and reducing power of the gastrointestinally digested CSPI were observed after one F–T cycle. The results suggest that the higher the polypeptide content was, the stronger the antioxidant activity. Hence, CSPI exhibited improved in vitro antioxidant activities after gastrointestinal digestion, which was similar to that of rice bran protein isolate [17]. Instead of decreasing the antioxidant activity of CSPI digestion, proper F–T cycles (1–3) may improve it.

## 4. Conclusions

Significant influences of multiple F–T cycles on CSPI structures, their functional properties, and IVGD were demonstrated in the research. During the F–T process, ice recrystallization and oxidized aggregation led to conformational changes and structural unfolding of the protein, thus contributing to variations in the content of free sulfhydryl and carbonyl groups. The above structural changes further influenced the IVGD and functional properties of CSPI. Nevertheless, the extent of influence depended on the F–T cycle number. Three F–T cycles enabled optimal emulsification performance of the CSPI emulsion. Moreover, 1–3 F–T cycles resulted in improved in vitro digestibility, polypeptide content of CSPI, and antioxidant activity of digestion. These findings further illustrate the effects of F–T cycles in modifying CSPI, improving emulsifying properties, and broadening the application of CSPI to food processing technologies.

## Data Availability

The data presented in this study are available on request from the corresponding author.
